# 3D Mapping of Safe and Danger Zones in the Maxilla and Mandible for the Placement of Intermaxillary Fixation Screws

**DOI:** 10.1371/journal.pone.0084202

**Published:** 2013-12-19

**Authors:** Kathiravan Purmal, Mohammad Khursheed Alam, Abdullah Pohchi, Noor Hayati Abdul Razak

**Affiliations:** 1 Oral and Maxillofacial Surgery Department, School of Dental Science, Universiti Sains Malaysia, Kota Bharu, Kelantan, Malaysia; 2 Orthodontic Unit, School of Dental Science, Universiti Sains Malaysia, Kota Bharu, Kelantan, Malaysia; INSERM U1059/LBTO, Université Jean Monnet, France

## Abstract

Intermaxillary (IMF) screws feature several advantages over other devices used for intermaxillary fixation, but using cone beam computed tomography (CBCT) scans to determine the safe and danger zones to place these devices for all patients can be expensive. This study aimed to determine the optimal interradicular and buccopalatal/buccolingual spaces for IMF screw placement in the maxilla and mandible. The CBCT volumetric data of 193 patients was used to generate transaxial slices between the second molar on the right to the second molar on the left in both arches. The mean interradicular and buccopalatal/buccolingual distances and standard deviation values were obtained at heights of 2, 5, 8 and 11 mm from the alveolar bone crest. An IMF screw with a diameter of 1.0 mm and length of 7 mm can be placed distal to the canines (2 - 11 mm from the alveolar crest) and less than 8 mm between the molars in the maxilla. In the mandible, the safest position is distal to the first premolar (more than 5 mm) and distal to the second premolar (more than 2 mm). There was a significant difference (p<0.05) between the right and left quadrants. The colour coding 3D template showed the safe and danger zones based on the mesiodistal, buccopalatal and buccolingual distances in the maxilla and mandible.The safest sites for IMF screw insertion in the maxilla were between the canines and first premolars and between the first and second molars. In the mandible, the safest sites were between the first and second premolars and between the second premolar and first molar. However, the IMF screw should not exceed 1.0 mm in diameter and 7 mm in length.

## Introduction

Intermaxillary fixation (IMF) screws are bicortical bone screws used to provide the upper and lower jaw in occlusion after trauma or orthognathic surgery. The benefits of using this screw instead of the traditional arch bars are the following: quick and easy procedure, compatibility with any plating system, no discomfort to the patient, reduced trauma to the buccal mucosa, ideal for use when the teeth have been heavily restored, gingival health is easier to maintain, reduced risk of sharp injury and easy removal [1]. The size of the screw is a diameter of 1.0 to 2 mm and length of 6 to 12 mm, and its composition is stainless steel or a titanium alloy.

Arthur and Berdardo [1] described an IMF screw as a single modality for the treatment of mandibular fractures. The first generation IMF screws were simply modified monocortical self-tapping screws [2]. They required a drilled hole for placement, and root damage occurred during their placement. The second generation self-drilling/-tapping screws improved the tactile feedback. The screws were recommended to be placed above the root apices by the manufacturers. However, such a high position will lead to irritation and mucosal overgrowth [3].

Although there are many benefits associated with the use of IMF screws, iatrogenic damage to the roots of teeth has been reported with their use [3,4]. Therefore, careful attention must be paid to the 3D relationship of the insertion path with the surrounding dental structures to reduce complications. Many studies have tried to identify the ideal placement area for orthodontic mini screws [5,6,7]. Poggio et al. [8] identified the safe positions for mini-implants to be used in orthodontics. These screws are almost the same as used in orthognathic or trauma except the screw head may vary in design. They evaluated the posterior quadrant in the mandible and maxilla and found that the least amount of bone was between the first premolar and canine and the most space was between the first and second molars. They concluded that the ideal screws should be 1.2 to 1.5 mm in diameter with a length of 6 to 8 mm. 

The prime aim of this study is to provide the surgeons with a guide for the safe placement of IMF screws by the 3D mapping of the safe and danger zones in the mesiodistal and buccolingual distances of all teeth at 2, 5, 8 and 11 mm from the alveolar crest of the maxilla and mandible.

## Methods

All participants provide their written informed consent prior cone beam computerised tomography (CBCT), and this study was approved by the Ethical Committee of the Hospital Universiti Sains Malaysia (HUSM), which complies with the Declaration of Helsinki. This study was designed and conducted according to the guidelines of Strengthening the Reporting of Observational studies in Epidemiology (STROBE), and we applied the STROBE checklist in the preparation of this manuscript [9]. 

The data source was CBCT (cone beam computerised tomography) volumetric data from the archives of the School of Dental Sciences, HUSM. A total of 104 measurements in each patient were recorded in 98 maxilla (63 male and 35 female), and a total of 104 measurements in each were recorded in 95 mandibles (47 male and 48 female).

Inclusion criteria

- Age between 20 and 50 years
- At least full dentition in one arch excluding the third molars
- High quality CBCT volumetric data
- Ethnicity verified from the folder.

Exclusion criteria

- Severe crowding
- Excessive spacing
- Radiographic evidence of pathology within the maxilla or mandible
- Periodontal disease
- Retained deciduous teeth.
- Fixed orthodontic appliance.

The CBCT data were acquired using Plameca Promax 3D (Helsinki, Finland). Plameca Romexis software was used to produce a secondary reconstruction of the volumetric data. Transaxial and sagittal slices (1 mm) were generated in the selected maxilla or mandibular images.

**Figure 1:**
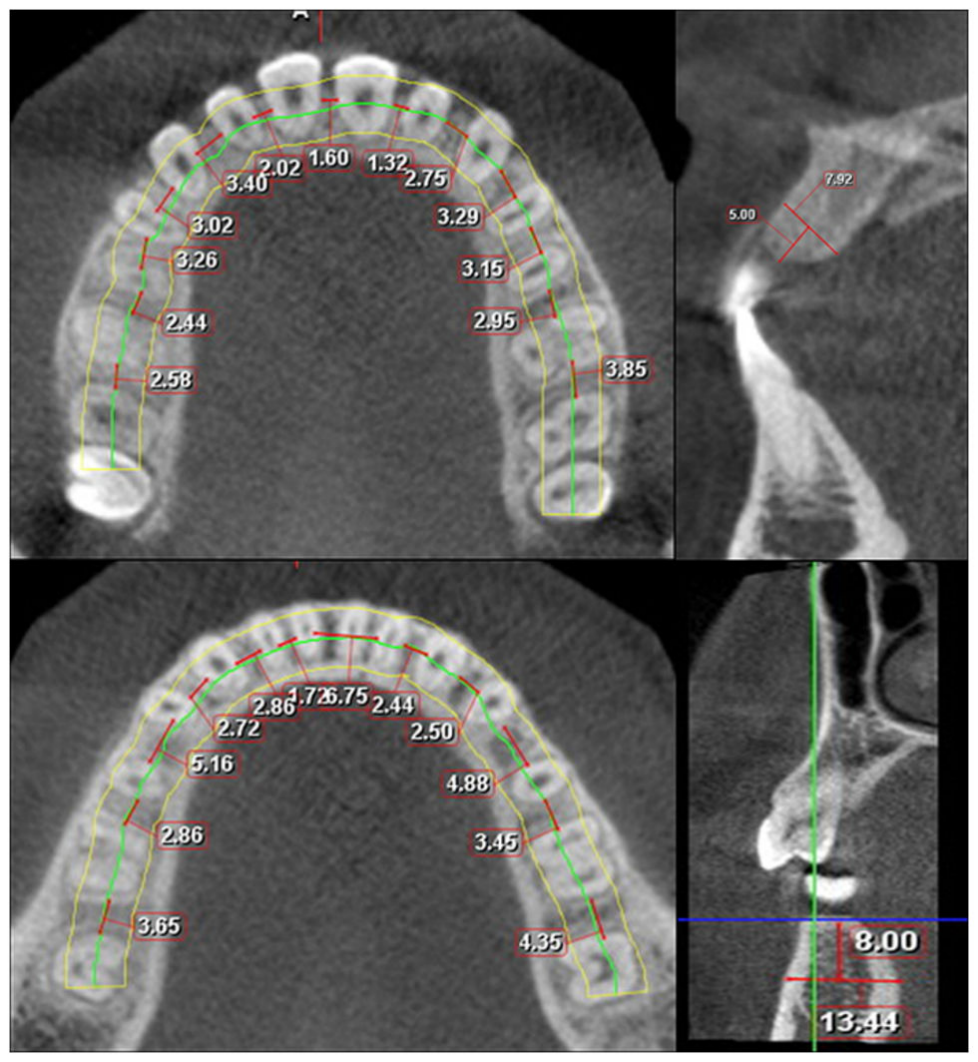
3D measurements of the mesiodistal distance in the maxilla (upper right) and mandible (lower right), buccopalatal distance in the maxilla (upper left) and buccolingual distance in the mandible (lower left).

Identical conditions were used for the measurement of the images throughout the study. The author performed a calibration periodically with a different set of CBCT images (not included in this study) with one supervisor. The linear measurements were made at depths of 2, 5, 8 and 11 mm with the alveolar crest as the reference point using Romexis version 2.6 software (8). The mesiodistal distance was measured parallel to the mean arch forms connecting the midroot portion of each root at each vertical level [10] (Figure **1**, upper and lower right). The buccolingual and buccopalatal distances were measured perpendicular to the mean arch form using the sagittal images constructed between the teeth at different depths from the alveolar crest at 2, 5, 8 and 11 mm (Figure **1**, upper and lower left).

### Statistical Analysis

The data were statistically analysed using SPSS version 20 (Chicago, USA). The normality of the data was evaluated with the skewness and kurtosis measurements. The means and standard deviations of the mesiodistal, buccolingual and buccopalatal dimensions at each vertical level were calculated. The paired t test was used to compare the mesiodistal, buccolingual and buccopalatal locations in the right and left quadrants. P-values less than 0.05 were considered to be statistically significant.

## Results

Calculations were made for 20 images that were randomly selected. The calculations were repeated again two weeks later. These calculations were performed to assess the systemic and random errors.

Systemic errors were measured using a two-sample t-test for each pair of readings. Houston [11] mentioned that there would be no systemic bias if the p value is greater than 0.1.

Random errors were estimated by calculating the correlation between repeated measurements (index of reliability). Stirrup [12] mentioned that a correlation value greater than 0.95 is acceptable. 

The skewness for all the variables was between + 1 and -1. The kurtosis, when divided by the standard error of kurtosis, produced a figure that was less than 1.96, indicating that the data were normally distributed.

All test and retest measurements showed an intra-class correlation of greater than 0.95, which is significant at 0.05. These results showed that there were no random errors.

All pairs of measurements showed a p value of greater than 0.1, which confirmed that there was no systemic bias in these readings.

The interradicular widths of the right and left sides of the maxilla and mandible at the various levels are shown in Table **1**. 

**Table 1:** Interradicular width of the right and left side of the maxilla and mandible at various heights.

				**Interradicular width** (**mm**)											
	Heights	1—1		1--2		2--3		3--4		4--5		5--6		6--7	
		Mean	SD	Mean	SD	Mean	SD	Mean	SD	Mean	SD	Mean	SD	Mean	SD
**Right Side**															
	2 mm	3.97	0.42	2.68*	0.41	2.17*	0.26	3.01*	0.44	3.00*	0.33	3.00	0.31	3.63*	0.32
**Maxilla**	5 mm	4.31	0.48	3.12*	0.42	2.60*	0.44	3.43	0.41	3.60*	0.49	3.36*	0.36	3.95*	0.49
	8 mm	4.61	0.49	3.39*	0.58	2.99*	0.46	3.85*	0.44	3.93	0.39	3.66*	0.36	4.16*	0.39
	11 mm	4.66	0.72	3.90	0.48	3.60*	0.47	4.24*	0.41	4.29	0.50	4.06*	0.37	4.56*	0.50
	2 mm	2.47	0.46	2.35*	0.26	2.33	0.17	2.28*	0.25	2.65	0.28	3.15*	0.13	4.08*	0.27
**Mandible**	5 mm	2.79	0.44	2.74*	0.34	2.76	0.23	2.81	0.28	3.27	0.26	3.60*	0.30	4.43*	0.31
	8 mm	3.07	0.41	2.99	0.33	3.32*	0.24	3.37	0.37	3.51*	0.41	4.09	0.32	5.03*	0.38
	11 mm	3.71	0.47	3.44	0.28	3.99*	0.32	4.01*	0.37	4.10*	0.35	4.84*	0.36	5.59*	0.39
**Left Side**	2 mm	2.36*	0.38	2.96*	0.33	3.07*	0.38	2.92*	0.36	3.21	0.63	2.70*	0.47	2.70*	0.47
**Maxilla**	5 mm	3.26*	0.51	3.61*	0.63	3.45	0.42	3.49*	0.33	3.31*	0.38	2.82*	0.47	2.82*	0.47
	8 mm	3.58*	0.51	3.78*	0.47	3.83*	0.38	3.69	0.29	3.81*	0.40	3.17*	0.55	3.17*	0.55
	11 mm	4.07	0.45	4.28*	0.48	4.26*	0.44	4.07	0.32	4.18*	0.43	3.83*	0.47	3.83*	0.47
	2 mm	2.07*	0.29	2.37	0.27	2.47*	0.29	2.57	0.29	2.53*	0.22	3.67*	0.36	3.67*	0.36
**Mandible**	5 mm	2.49*	0.30	2.83	0.36	2.91	0.36	3.20	0.28	3.30*	0.38	4.33*	0.40	4.33*	0.40
	8 mm	2.96	0.36	3.17*	0.46	3.43	0.38	3.66*	0.29	4.02	0.42	4.72*	0.30	4.72*	0.30
	11 mm	3.37	0.36	3.55*	0.42	3.91*	0.31	4.02*	0.26	4.68*	0.40	5.12*	0.19	5.12*	0.19

* shows p< 0.05 (significant difference between right and left side).

Only five pairs of readings showed no significant difference between the right and left quadrants of the maxilla. Nineteen pairs showed a significant difference at the 0.05 level. Therefore, there was a significant difference between the right and left quadrants with respect to the mesiodistal measurement. 

The majority of the readings (15 pairs) showed no significant difference (p>0.05) between the right and left quadrants in the mesiodistal measurement of the mandible. This trend is similar to that of the maxilla, where most of the differences were less than 0.5 mm.

The buccopalatal and buccolingual widths of the right and left sides of the maxilla and mandible at various levels are shown in Table **2**. 

**Table 2:** Buccopalatal and buccolingual width of the right and left side of the maxilla and mandible at various heights.

				**Buccopalatal and buccolingual width** (**mm**)											
	Heights	1—1		1--2		2--3		3--4		4--5		5--6		6--7	
		Mean	SD	Mean	SD	Mean	SD	Mean	SD	Mean	SD	Mean	SD	Mean	SD
**Right Side**															
	2 mm	4.34	0.7	6.48*	0.74	5.95	0.67	8.23*	0.80	8.65*	0.85	10.48	0.90	12.71*	1.49
**Maxilla**	5 mm	4.91	0.67	6.73*	0.82	6.22*	0.86	8.28*	0.89	8.84*	0.86	10.37	1.11	12.66*	1.41
	8 mm	5.58	0.85	7.07*	0.98	6.65*	0.87	8.57*	1.19	8.79*	1.14	10.15*	1.67	3.58*	2.93
	11 mm	6.10	0.92	7.62*	0.84	7.32*	0.92	9.02*	1.44	9.17*	1.35	3.34*	2.45	2.71	2.64
	2 mm	5.99	1.86	5.91*	1.45	6.97	1.23	7.27*	1.40	7.82	2.00	8.72*	1.26	10.94*	1.29
**Mandible**	5 mm	6.20	1.22	6.29*	1.54	7.30	0.92	7.88	1.19	8.42*	1.83	9.30*	1.09	11.68*	1.30
	8 mm	6.77	2.01	6.94	1.70	7.83*	1.37	8.53	1.36	9.03*	1.99	9.86	1.23	12.22*	1.18
	11 mm	7.71	1.77	7.74*	1.62	8.25*	1.41	9.29*	1.68	9.79*	1.98	10.86*	1.11	12.79*	1.30
**Left Side**	2 mm	4.88*	0.73	5.74	0.75	6.75*	0.73	5.86*	1.17	10.57	1.16	11.64*	1.46	4.88*	0.73
**Maxilla**	5 mm	5.11*	0.81	5.92*	0.94	7.01*	0.74	6.39*	1.02	10.30	1.17	10.92*	2.51	5.11*	0.81
	8 mm	5.38*	0.74	6.26*	1.05	7.37*	0.79	6.75*	0.91	4.94*	2.01	4.61*	3.19	5.38*	0.74
	11 mm	5.77*	0.93	6.81*	1.11	7.82*	1.12	7.20*	1.00	3.81*	2.29	2.98	2.68	5.77*	0.93
	2 mm	5.64*	1.06	6.59	1.49	8.25*	1.39	8.54	1.60	10.16*	1.98	10.66*	1.15	5.64*	1.06
**Mandible**	5 mm	6.06*	1.22	7.35	1.35	8.88	1.38	9.20*	1.51	10.68*	1.90	11.22*	1.18	6.06*	1.22
	8 mm	6.89	1.21	7.95*	1.58	9.48	1.41	9.67*	1.44	11.36	1.61	11.92*	1.15	6.89	1.21
	11 mm	7.59*	1.45	8.63*	1.45	10.10*	1.56	10.44*	1.56	11.82*	1.55	12.49*	1.07	7.59*	1.45

* shows p< 0.05 (significant difference between right and left side)

The p value for the buccopalatal distance was greater than 0.05 in only four of the readings, demonstrating that most of the readings were significantly different between the right and left sides. These differences were also larger than the mesiodistal widths. The maximum difference was (5.20±2.79) mm between the first molars and first premolars at the level 8 mm from the alveolar crest.

The majority of the measurements showed that there was a significant difference between the right and left quadrants (19 pairs) for the buccolingual distance. Only five readings had p>0.05, showing no significant difference between the right and left quadrants.

Table **3** compares the results of various studies [8,5,13,14,15,16,17] with the present study. The sample size were limited in the other studies (12-25 participants) compared to our study which had 98 (maxilla) and 95 (mandible). 

**Table 3:** Global and present study data regarding the safest sites for the insertion of IMF screws in the maxilla and mandible.

**Measures Between Tooth-Location From The Bone Crest**																					
Studies (Sample size)		7—6				6--5				5--4				4--3				3--2			
		0-3 mm	3-6 mm	6-9 mm	9-12 mm	0-3 mm	3-6 mm	6-9 mm	9-12 mm	0-3 mm	3-6 mm	6-9 mm	9-12 mm	0-3 mm	3-6 mm	6-9 mm	9-12 mm	0-3 mm	3-6 mm	6-9 mm	9-12 mm
**MAXILLA**	Poggio et al, 2006 (25)	2.5	2.3	2.5	0.8	2.7	2.9	3.0	1.6	2.9	3.2	3.5	3.3	3.0	3.4	3.9	4.3	nil	nil	nil	nil
	Degushi et al,2006 (10)	nil	1.5	3.8	nil	nil	2.1	6.1	nil	nil	nil	nil	nil	nil	nil	nil	nil	nil	nil	nil	nil
	Bittencourt et al, 2011 (12)	1.2	1.0	1.0	1.5	1.7	1.7	1.9	2.0	1.4	1.4	1.5	2.0	1.6	1.7	2.1	2.7	1.3	1.6	1.6	nil
	Hernandez et al , 2008 (21)	4.0	3.0	5.0	nil	3.0	3.0	4.0	nil	3.0	3.0	4.0	nil	3.0	3.0	4.0	nil	2.0	2.0	3.5	nil
	Silvestrini Biavati et al, 2011 (25)	nil	2.0	2.2	2.4	nil	2.7	3.0	3.6	nil	2.6	2.7	2.8	nil	2.4	2.6	2.8	nil	nil	nil	nil
	Present study (98)	3.2	3.1	3.9	4.3	3.1	3.5	3.7	4.2	2.9	3.5	3.9	4.3	3.0	3.5	3.8	4.2	2.6	3.0	3.4	3.9
**MANDIBLLE**	Poggio et al, 2006 (25)	3.2	3.0	3.5	4.7	3.0	2.9	3.1	3.9	3.2	3.7	4.3	4.9	2.7	2.8	3.0	3.5	nil	nil	nil	nil
	Monnerat et al, 2009 (13)	3.7	4.1	4.9	6.2	3.0	3.2	3.9	4.5	2.6	3.0	3.7	3.9	1.9	2.1	2.4	2.8	1.8	2.1	2.6	3.0
	Bittencourt et al, 2011 (12)	2.5	2.7	3.3	4.4	1.8	2.0	2.2	2.5	2.9	3.5	1.2	3.4	1.7	1.7	1.1	1.7	2.8	2.6	nil	nil
	Park and Cho, 2009 (21)	nil	1.6	1.6	2.0	nil	2.4	2.7	3.3	nil	2.0	2.2	2.4	nil	2.2	2.4	2.6	nil	nil	nil	nil
	Present study (95)	3.9	4.4	4.9	5.4	2.8	3.5	4.1	4.8	2.6	3.2	3.6	4.1	2.5	2.9	3.4	4.0	2.4	2.8	3.2	3.8

In the maxilla, our results were comparable with Hernandez et al [14]. The differences were between 0.0 to 1.2mm only. The most differences were with studies by Bittencourt et al [13] (1.3 to 2.9mm). The trend of increasing distance towards the apical position is similar in all the studies except in the study by Poggio et al [8] where there is a reduction from 2.5mm to 0.8 mm in-between the upper 1^st^ molar and 2^nd^ molar from the level of 6-9 mm to 9-12mm.

In the mandible, our results were close to Monnerat et al [16] study. The differences were between 0 to 1.2mm only. However the trends of increasing distance towards the apex were similar in all the studies.

**Figure 2:**
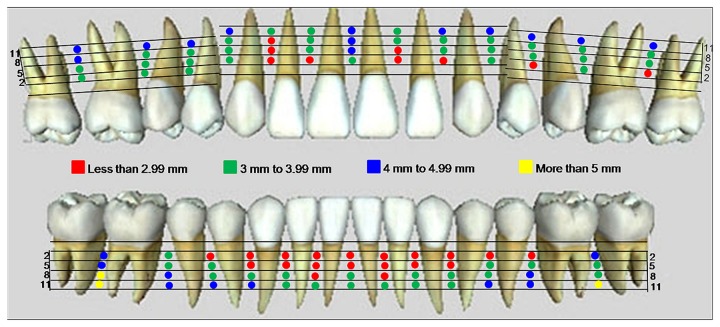
3D anatomical mapping for safe and danger zones based on the mesiodistal distance in the maxilla and mandible. The red areas indicate danger zones, and the green, blue and yellow areas indicate the safe zones.

**Figure 3:**
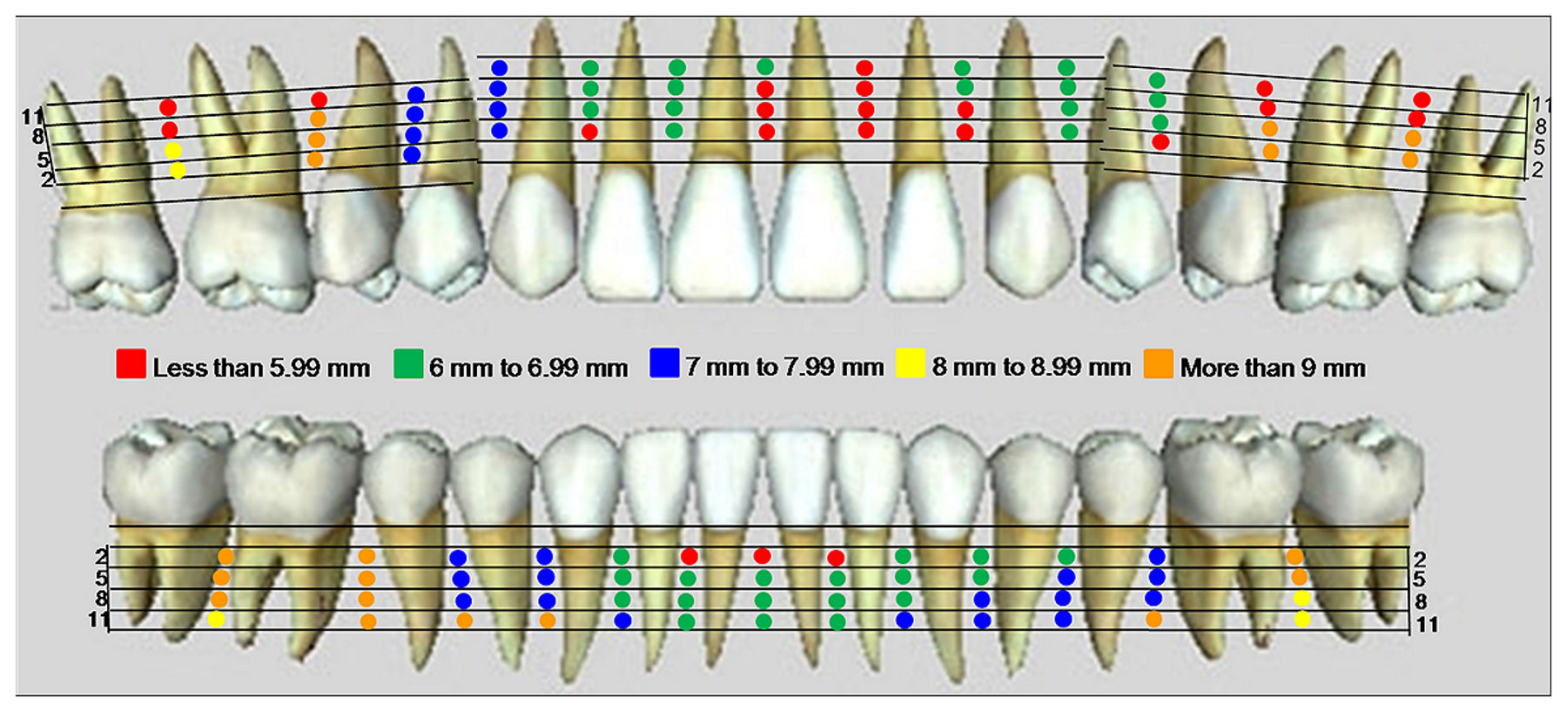
3D anatomical mapping for safe and danger zones based on the buccopalatal distance in the maxilla and buccolingual distance in the mandible. The red areas indicate danger zones, and the green, blue, yellow and orange areas indicate the safe zones.

Figure **2** shows the mesiodistal width of the interradicular distance in the maxilla and mandible. Figure **3** shows the buccopalatal distance in the maxilla and buccolingual distance in the mandible. Different colours represent the safe and danger zones for IMF screw insertion. 

## Discussion

According to Houston [11], the most important contributions to the improvement of landmark identification are experience and calibration. Therefore, the author performed periodic calibrations with a different set of CBCT images (not included in this study) with one of the supervisors. Only one experienced radiographer was responsible for taking the images in the same position as specified by the manufacturer of the radiographic equipment. 

There has been a dramatic increase in the use of CBCT in dentistry over the last decade. This technology has found particular applications for diagnosis and treatment planning in both adult and paediatric patients [18]. The CBCT data can be reconstructed to provide unique images previously unavailable in clinical practice. Innately, CBCT data are presented as inter-relational undistorted images in three orthogonal planes (axial, sagittal, and coronal); however, software techniques are readily available (maximum intensity projection and surface or volumetric rendering) to provide a three-dimensional visualisation of the maxillofacial skeleton [19]. 

Each location in the dental alveolus has unique morphologic characteristics due to edentulousness and specific regional anatomic features that need to be identified and assessed in the diagnostic and planning phases for the treatment with IMF screws. 

There were significant side differences among the interradicular, buccolingual and buccopalatal distances in both arches (Table **1** and **2**). There is currently no other research that has reported this observation. Most authors have assumed both sides to be symmetrical and consequently measured either the right or left side of the quadrant only [8,10,20]. The possible explanations for this discrepancy maybe related to the musculature. The cortical bone thickness is influenced by the muscles involved in mastication [21]. The Caucasian population has been found to have the greatest amount of interdental bone in the maxillary buccal region between the second premolar and first molar 5 to 8 mm apical to the alveolar crest [8,22,23]. There are differences between Caucasian and Asian tooth morphology [24], which can influence the amount of interdental bone that is available for IMF screw insertion. Ethnic differences in the tooth root length and crown size and shape are also well documented [25,26].

Based on Figure **2**, in the maxilla, the area to avoid screw placement, identified in red (less than 3 mm), is found between the lateral incisor and canine 5 mm from the alveolar crest on the left side and 8 mm from the alveolar crest on the right side. This difference could be due to the distal inclination and curvature of the lateral incisor root. Most other areas are noted as green, which is between 3 and 4 mm. These green areas represent safe zones for the insertion of an IMF screw with a diameter of 1.5 mm. The blue areas are located mainly in the 11 mm distance from the alveolar crest, except for between the central incisors. 

The location of mucogingival junction was not measured in our subjects. Therefore we reviewed the literature [27] to find the normative value for attached gingiva which is 3 to 5 mm apical to the level of crestal bone.

As per Figure 1, safe area with attached gingival (less 5mm from the alveolar bone) would be in-between cental incisors, distal of canines, premolars and molars in the upper arch.

In the lower arch, the safe area with attached gingival are distal of second premolars in the right and in-between the molars bilaterally. All the other safe area will be in mobile mucosa. In order to avoid irritation from the mobile mucosa, Rai et al [28] suggested a modified screw head which can be used when the IMF screw is placed above 5 mm from the alveolar crest.

There is a limited interradicular distance for IMF screw insertion in the mandible, as shown in Figure **2**. The red area (less than 3 mm) is located between the central incisors up to 5 mm from the alveolar crest. The narrowest area is between the lateral and central incisors, representing a danger zone up to 8 mm from the alveolar crest. Mesiodistal to the canine, space is available at the level of 5 mm from the alveolar crest. The maximum space is found between the first and second molars and more than 8 mm from the alveolar crest. In Figure **3**, the buccopalatal distance is evaluated. In the maxilla, the red area, which is less than 6 mm, is mainly due to the anatomical landmarks such as the maxillary sinus and nasopalatine foramen. The area on the left between the central and lateral incisors also showed a reduced buccopalatal space. Therefore, IMF screws longer than 6 mm should not be used in those areas. The maximum space is available between the first premolar and first molar up to 8 mm from the alveolar crest.

In the anterior part of the mandible (Figure **3**), the distance is between 6 and 7 mm, as shown by the green area. Therefore, a long IMF screw should be used in the region distal to the canines. The maximum buccolingual distance is between the first and second molars.

Data from other population studies (Table **3**) [8,5,13,14,15,16,17] confirmed the trend of increasing the distance as the measurements are taken farther from the alveolar crest in both arches. The differences in the values between our and the other studies may be due to the racial mix, confirming that each race should have their own set of data. 

This study analysed the mesiodistal, buccopalatal, and buccolingual positions for the safe insertion of IMF screws. For the clinical evaluation of the data, it is crucial to interrelate the measurements with IMF screw diameter and the minimal bone clearance needed for periodontal health and screw stability. The width of the periodontal ligament is known to be approximately 0.25 mm [29]. The IMF screw is 1 to 2 mm in diameter and 6 to 12 mm in length. Therefore, the minimum mesiodistal width necessary would be 3 mm for a 1 mm diameter screw and 4 mm for a 2 mm diameter screw [22]. In both the maxilla and mandible, the mesiodistal interradicular measurements were less than the buccolingual/buccopalatal measurements. Therefore, the mesiodistal interradicular measurements are the key parameters to define the interradicular space suitable for IMF screw insertion. 

We developed a template through the 3D analysis of 104 measurements for 98 maxilla and 104 measurements for 95 mandibles. Our template presented the 3D mapping of the safe and danger zones in the maxilla and mandible for the placement of IMF screws with colour coding in a simplified manner for the ease of application. These findings, a using 3D analysis, were obtained from Malay subjects at HUSM. Whether similar findings might be obtained in another population is unknown. Conducting this 3D analysis in study populations from other institutions might be useful.

## Conclusions

The methodology used in this study and a review of the relevant literature has led us to conclude that the best sites for the placement of IMF screws are the following:

1. In the maxilla, distal to the canines (2 - 11 mm from the alveolar crest) and less than 8 mm between the first and second molars.
2. In the mandible, distal to the first premolar (more than 5 mm) and distal to the second premolar (more than 2 mm).
